# Association between cancer stem cell gene expression signatures and prognosis in head and neck squamous cell carcinoma

**DOI:** 10.1186/s12885-022-10184-4

**Published:** 2022-10-19

**Authors:** Su Il Kim, Seon Rang Woo, Joo Kyung Noh, Min Kyeong Lee, Young Chan Lee, Jung Woo Lee, Moonkyoo Kong, Seong-Gyu Ko, Young-Gyu Eun

**Affiliations:** 1 Department of Otolaryngology-Head and Neck Surgery, Kyung Hee University Medical Center, #1 Hoegi-dong, Dongdaemun-gu, Seoul, 02447 Korea; 2 Department of Biomedical Science and Technology, Graduate School, Kyung Hee University, Seoul, Korea; 3 Department of Oral and Maxillofacial Surgery, School of Dentistry, Kyung Hee University, Seoul, Korea; 4 Department of Radiation Oncology, Division of Lung & Head and Neck Oncology, Kyung Hee University Medical Center, Seoul, Korea; 5 Department of Preventive Medicine, College of Korean Medicine, Kyung Hee University, Seoul, Korea

**Keywords:** Head and neck squamous cell carcinoma, Cancer stem cell, Gene expression signature, Overall survival, Recurrence-free survival

## Abstract

**Background:**

Various cancer stem cell (CSC) biomarkers and the genes encoding them in head and neck squamous cell carcinoma (HNSCC) have been identified and evaluated. However, the validity of these factors in the prognosis of HNSCC has been questioned and remains unclear. In this study, we examined the clinical significance of CSC biomarker genes in HNSCC, using five publicly available HNSCC cohorts.

**Methods:**

To predict the prognosis of patients with HNSCC, we developed and validated the expression signatures of CSC biomarker genes whose mRNA expression levels correlated with at least one of the four CSC genes (*CD44*, *MET*, *ALDH1A1*, and *BMI1*).

**Results:**

Patients in The Cancer Genome Atlas (TCGA) HNSCC cohort were classified into CSC gene expression-associated high-risk (CSC-HR; *n* = 285) and CSC gene expression-associated low-risk (CSC-LR; *n* = 281) subgroups. The 5-year overall survival and recurrence-free survival rates were significantly lower in the CSC-HR subgroup than in the CSC-LR subgroup (*p* = 0.04 and 0.02, respectively). The clinical significance of the CSC gene expression signature was validated using four independent cohorts. Analysis using Cox proportional hazards models showed that the CSC gene expression signature was an independent prognostic factor of non-oropharyngeal HNSCC which mostly indicates HPV (–) status. Furthermore, the CSC gene expression signature was associated with the prognosis of HNSCC patients who received radiotherapy.

**Conclusion:**

The CSC gene expression signature is associated with the prognosis of HNSCC and may help in personalized treatments for patients with HNSCC, especially in cases with HPV (–) status who were classified in more detail.

**Supplementary Information:**

The online version contains supplementary material available at 10.1186/s12885-022-10184-4.

## Background

Head and neck squamous cell carcinoma (HNSCC) is the sixth most common cancer worldwide and includes all cancers that occur in the mucosa of the oropharynx, oral cavity, hypopharynx, or larynx [1]. Approximately 650,000 new cases of HNSCC occur every year and 350,000 patients die of it worldwide [2]. Despite advances in therapeutic methods, the survival rates of this condition have not markedly improved over the past few decades [3].

HPV status is a well-known factor that influences the prognosis of patients with HNSCC [4]. Recently, various molecular markers that influence HNSCC prognosis have been identified for precision medicine and personalized treatment [5]. However, there are many other molecular markers that require further investigation. Cancer stem cells (CSCs) and CSC markers are important targets in this respect [6].

CSCs constitute the part of a tumor that has long-term repopulation potential, the ability to evade cell death, clonal tumor initiation capacity, and self-renewal properties [7]. CSCs have been identified by their cell surface markers expression, which are mostly selected by embryonic stem cells or by self-properties involved with tissue development lineage molecules [8]. The most well-known CSC marker is CD44, which is associated with cell proliferation, angiogenesis, adhesion, and migration during tumorigenesis [9]. In addition to CD44, 27 other CSC biomarkers have been reported and evaluated in HNSCC [10].

However, CSCs comprise only a small proportion of cancer cells [11] and can be regulated by external forces and cell-autonomous forces [12]. In other words, CSCs may not necessarily be rare within tumors, and non-CSCs in tumors can be reversibly reprogrammed to become CSCs [13]. Thus, genes encoding CSC markers in tumors need to be analyzed, regardless of whether they are CSCs or non-CSCs. However, the validity and clinical significance of these genes have been questioned recently and remain to be ascertained in the context of HNSCC. Additionally, the validity and underlying relationship among these CSC biomarker genes in predicting the prognosis of HNSCC have not been demonstrated. Therefore, further research is needed to validate the role of CSC biomarker genes in HNSCC and determine personalized treatments for patients with HNSCC.

In this study, we analyzed the genomic data of patients with HNSCC to determine the molecular subtypes associated with CSC biomarker genes, thereby predicting their prognosis. We hypothesized that the investigation of mRNA expression of various genes including CSC biomarker genes in The Cancer Genome Atlas (TCGA) HNSCC cohort would generate CSC gene expression-associated molecular signatures, which could be validated in various independent HNSCC cohorts. We also investigated the prognostic importance of CSC gene expression signatures in various subgroups of patients with HNSCC.

## Methods

### Patient cohorts

Gene expression levels and clinical data from five independent cohorts were downloaded from public databases. Using the UCSC Cancer Genomics Browser (https://xena.ucsc.edu/public), clinical and gene expression data of TCGA cohort (*n* = 566) were obtained. Using the National Center for Biotechnology Information Gene Expression Omnibus database (http://www.ncbi.nlm.nih.gov/geo), corresponding data from the Institute for Medical Informatics, Statistics and Epidemiology (Leipzig cohort, GSE65858, *n* = 270) [14], Fred Hutchinson Cancer Research Center (FHCRC cohort, GSE41613, *n* = 97) [15], MD Anderson Cancer Center (MDACC cohort, GSE42743, *n* = 74) [15], and AHEPA Hospital in Thessaloniki (Greece cohort, GSE27020, *n* = 109) [16] were obtained. The gene expression profile of TCGA cohort was measured using Illumina HiSeq® 2000 (Illumina Inc., San Diego, CA, USA), while that of the Leipzig cohort was measured using Illumina HumanHT-12 v4.0 Expression BeadChip, and those of the FHCRC, MDACC, and Greece cohorts were measured using Affymetrix Human Genome U133 Plus 2.0 Array (Affymetrix Inc., California, USA). All gene expression data were standardized using different platforms.

### Selection of reference CSC genes (training cohort)

The search results for the reported CSC biomarkers and encoding genes were obtained from the study by Xiao et al. [10]. Specifically, CSC biomarkers and encoding genes were searched for in PubMed, using the terms ‘tumor stem cells’, ‘tumor stem-like cells’, ‘CSCs’, ‘cancer stem cells’, ‘cancer stem-like cells’, and ‘HNSCC’, ‘head and neck squamous cell carcinoma’. Twenty-eight genes were selected from the studies that met the following criteria: 1) studies in humans, 2) studies showing validated evidence, and 3) an original research paper. Case reports, comments, reviews, letters to the editor, and conference abstracts were excluded.

To select reference CSC genes among the 28 CSC genes, we additionally searched for articles written between 2012 and 2021 on CSC biomarkers or encoding genes in HNSCC. Articles were searched for in PubMed, using the terms ‘HNSCC’, ‘head and neck squamous cell carcinoma’, and each term about ‘CSC biomarkers and encoding genes’. We selected genes that satisfied the following criteria: (a) CSC biomarkers showing clinical significance when classified according to expression and (b) those that have been studied more than twice.

The selected genes were analyzed using a training cohort (TCGA cohort). First, TCGA cohorts were classified into two subgroups based on the mRNA expression of each gene. To define dichotomous cut-off values for continuous mRNA expression for each gene, an online tool (http://molpath.charite.de/cutoff/) was used [17]. The Kaplan–Meier method was used to generate survival curves for each subgroup of each gene. The log-rank test was used to compare the prognoses of the two subgroups for each gene. CSC genes showing significant differences in the 5-year overall survival (OS) or recurrence-free survival (RFS) rates between the two subgroups classified according to the mRNA expression were selected as reference CSC genes.

### Development of CSC gene expression-associated signature

Gene expression data from TCGA cohort were analyzed to identify CSC gene expression-associated signatures in HNSCC. Genes whose mRNA expression levels were negatively or positively correlated with at least one CSC gene marker were selected. We then performed unsupervised hierarchical clustering analysis with the centered correlation coefficient as a measure of similarity and a complete linkage clustering method using the Gene Cluster 3.0 program (Stanford University, Stanford, CA, USA; downloaded at https://cluster2.software.informer.com) [18]. In detail, selected CSC gene markers were adjusted, checking center genes with median methods. Next, adjusted data were divided into two groups using unsupervised hierarchical clustering, checking genes and arrays cluster with the centered correlation, and a complete linkage clustering method. The patients were divided into CSC gene expression-associated high-risk (CSC-HR) and low-risk (CSC-LR) subgroups. The subgroup showing significantly lower survival rates than the other group was defined as the CSC-HR subgroup. The Java Treeview program was used to generate heat maps for the cluster analysis.

### Construction of prediction models and validation in the four independent cohorts

Before constructing the prediction models, all gene expression data for each cohort were standardized by being transformed into a median of 0 and standard deviation of 1 because they were generated using different platforms. The Support Vector Machine (SVM) class prediction engine was used to test the ability of CSC gene expression-associated signatures to predict the class of patients with HNSCC in four independent cohorts [19]. Gene expression data from TCGA cohort were combined to form a series of classifiers according to the SVM algorithm, following which the robustness of the classifier was estimated according to the misclassification rate determined during leave-one-out cross-validation of the training set using BRB-Array Tools [20]. The validation was conducted in four independent cohorts (Leipzig, FHCRC, MDACC, and Greece).

### Pathway analysis

To identify gene ontology categories with significantly enriched gene numbers, 81 CSC gene expression-associated signatures were analyzed using the Database for Annotation, Visualization, and Integrated Discovery (DAVID) (version 6.8) [21]. To map the CSC gene expression signature to the reference set of direct and indirect relationships, default settings from the software were utilized. Relevant inputs to the gene list, such as biological functions and molecular networks, were then generated using the software’s algorithm. Significant gene annotation was determined using a two-tailed Fisher’s exact test (*p* < 0.05).

### Statistical analysis

Gene expression and survival data were used to test prognostic significance. OS was defined as the number of months between the date of diagnosis and the date of death. The number of months from the date of diagnosis to recurrence was defined as the RFS. The Kaplan–Meier method was used to produce OS and RFS curves in each subgroup of each cohort. The log-rank test was used to compare the OS and RFS rates between each subgroup. Receiver operating characteristic (ROC) curves were used to compare the sensitivity and specificity of the 1-year, 3-year, and 5-year survival predictions of CSC gene expression signatures. The area under the curve (AUC) was calculated for each ROC curve. Univariate and multivariate Cox regression models were used to evaluate the independent prognostic factors associated with the survival of patients with HNSCC. The results of the Cox regression analyses were reported as hazard ratios (HRs), 95% confidence intervals (95% CIs), and p-values. The R software package (http://www.r-project.org) was used for all statistical analyses. Statistical significance was set at *p* < 0.05.

## Results

### Development of CSC gene expression-associated signatures in patients with HNSCC

Among the 28 CSC genes encoding CSC biomarker proteins that have been validated in HNSCC [10], we selected seven CSC genes that satisfied the following criteria, *CD44*, *MET*, *ALDH1A1*, *BMI1*, *PROM1*, *SOX2*, and *POU5F1* from a literature search: (a) showing clinical significance associated with the expression of corresponding CSC biomarker proteins in HNSCC and (b) studied more than twice over the past 10 years [5, 8, 9, 22–30]. In TCGA cohort, high expression of four CSC genes (*CD44*, *MET*, *ALDH1A1*, and *BMI1*) was significantly associated with patient prognosis (*p* = 0.0069, 0.0051, 0.028, and 0.021, respectively; Fig. S1). Thus, these four genes were selected as the reference CSC genes for this study.

**Fig. 1:**
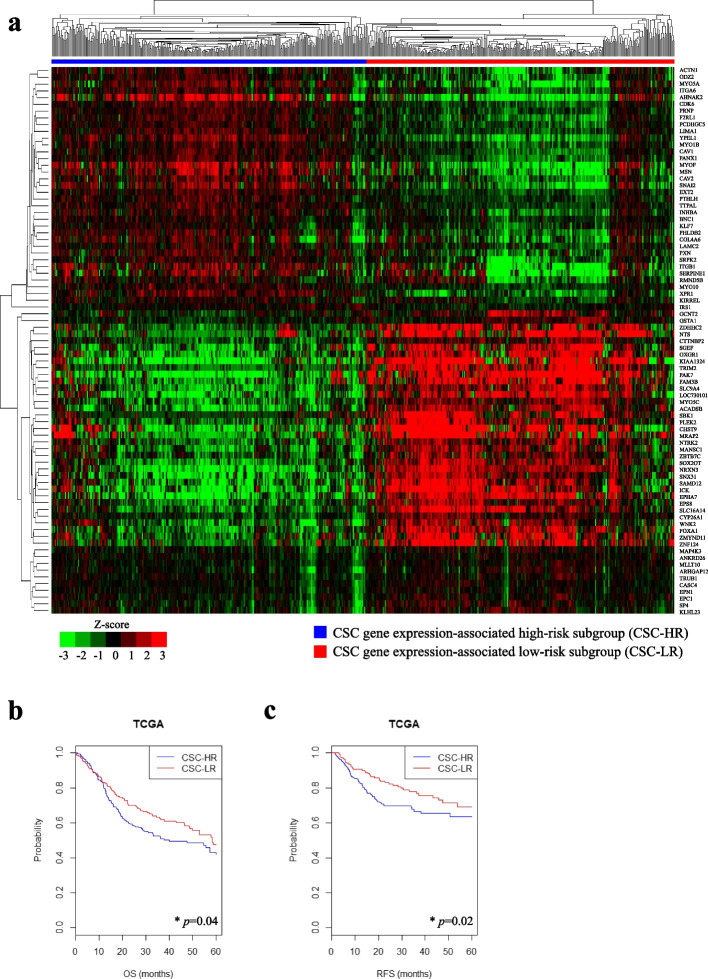
Stratification of patients with TCGA HNSCC cohort according to the 81 CSC gene expression signatures. **a** Patients from TCGA HNSCC cohort were classified into CSC-HR (*n* = 285) and CSC-LR (*n* = 281) subgroups, by means of hierarchical clustering. **b**-**c** The 5-year OS and RFS rates of each group were determined using Kaplan–Meier plots. The CSC-HR subgroup presented significantly lower 5-year OS and RFS rates than those of the CSC-LR subgroup (*p* = 0.04 and 0.02, respectively). *p < 0.05

We then identified genes whose mRNA expression was correlated with at least one of the four reference CSC genes in TCGA cohort. A total of 81 genes were identified and selected as CSC gene expression-associated signatures (Fig. S2a and Table S1). Using the CSC gene expression signatures, patients in TCGA cohort (*n* = 566) were classified into the CSC-HR (*n* = 285) and CSC-LR (*n* = 281) subgroups (Fig. 1a). The mRNA expression levels of *CD44* and *MET* were significantly higher in the CSC-HR subgroup than in the CSC-LR subgroup (*p* < 0.0001 both). The mRNA expression levels of *ALDH1A1* and *BMI1* were significantly higher in the CSC-LR subgroup than in the CSC-HR subgroup (*p* < 0.0001 both). The results of the Kaplan–Meier analysis and log-rank test indicated that the 5-year OS and RFS rates were significantly lower in the CSC-HR subgroup than in the CSC-LR subgroup, in TCGA cohort (*p* = 0.04 and 0.02, respectively; Fig. 1b-c).

### Independent validation of the CSC gene expression-associated signature

**Fig. 2:**
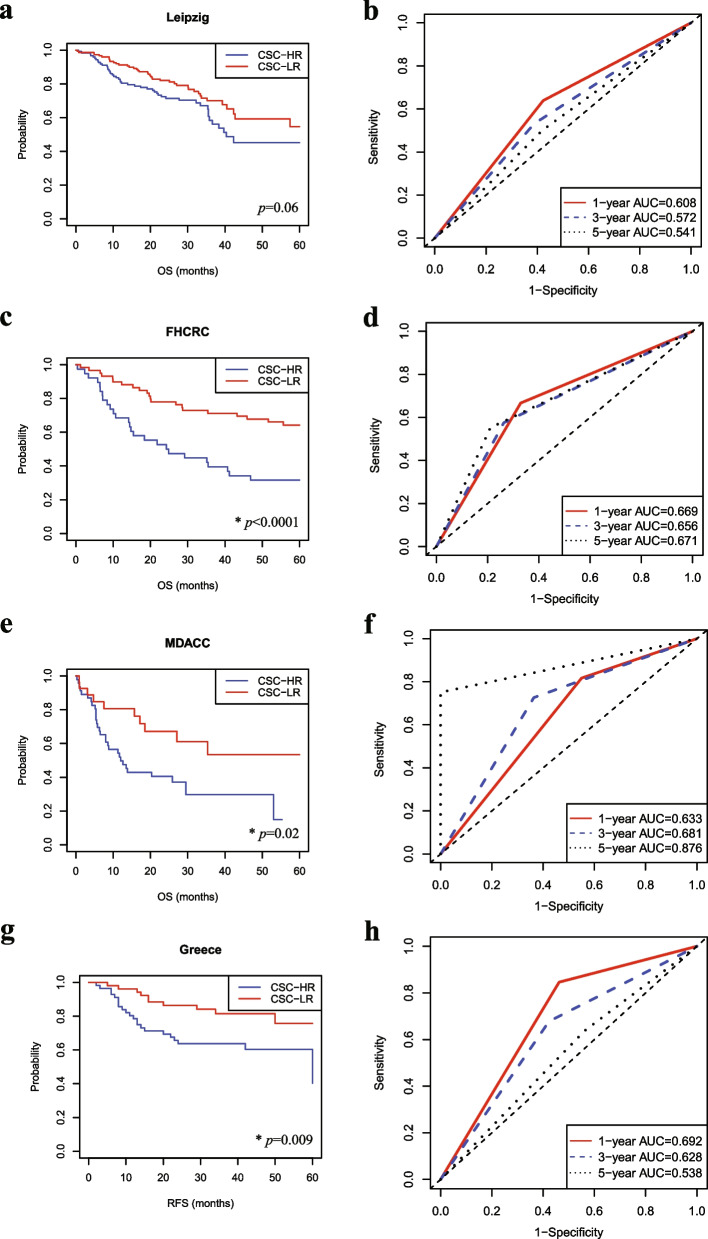
Validation of the 81 CSC gene expression signatures in the four independent HNSCC cohorts. Each cohort was stratified into CSC-HR and CSC-LR subgroups, on the basis of the 81 CSC gene expression signatures. The predicted outcomes in the four cohorts were assessed using Kaplan–Meier plots and ROC curves. **a**, **c**, **e** The 5-year OS rates of each subgroup were determined in the Leipzig (*n* = 270), FHCRC (*n* = 97), and MDACC (*n* = 109) cohorts (*p* = 0.06, < 0.0001, and 0.02, respectively). **b**, **d**, **f** The ROC curves show the sensitivity and specificity of the CSC gene expression signatures in predicting 1-year, 3-year, and 5-year patient OS rates in the Leipzig, FHCRC, and MDACC cohorts (AUC = 0.541, 0.671, and 0.876 for the 5-year OS, respectively). **g** The 5-year RFS rates of each subgroup was determined in the Greece cohort (*n* = 109; *p* = 0.009). **h** ROC curves show the sensitivity and specificity of the CSC gene expression signatures in predicting 1-year, 3-year, and 5-year patient RFS rates in the Greece cohorts (AUC = 0.538 for the 5-year RFS). *p < 0.05

The CSC gene expression signature was validated using four independent cohorts: Leipzig (*n* = 270), FHCRC (*n* = 97), MDACC (*n* = 74), and Greece (*n* = 109) (Fig. S2b). Details of the clinical and pathological characteristics of each cohort used in this study are shown in Table 1. Patients in each validation cohort were efficiently classified into CSC-HR and CSC-LR subgroups, based on the CSC gene expression signature. The CSC-HR subgroup in each validation cohort had a worse prognosis than the CSC-LR subgroup (Fig. 2). Five-year OS rates tended to be lower in the CSC-HR subgroup than in the CSC-LR subgroup, in the Leipzig cohort, although the differences were not significant (*p* = 0.06; Fig. 2a). In the FHCRC and MDACC cohorts, the 5-year OS rates were significantly lower in the CSC-HR subgroup than in the CSC-LR subgroup (*p* < 0.0001 and = 0.02, respectively; Fig. 2c and e). Furthermore, the 5-year RFS rates were significantly lower in the CSC-HR subgroup than in the CSC-LR subgroup, in the Greece cohort (*p* = 0.009; Fig. 2g). In all patients in the five cohorts, the 5-year OS rates were significantly lower in the CSC-HR subgroups than in the CSC-LR subgroups (*p* < 0.0001; Fig. S3a).

**Table 1:** Clinical and pathological characteristics of the five independent HNSCC cohorts^a^

Characteristics	TCGA cohort (*n* = 566)	Leipzig cohort (*n* = 270)	FHCRC cohort (*n* = 97)	MDACC cohort (*n* = 74)	Greece cohort (*n* = 109)
Age					
≥ 60	316 (55.83%)	117 (43.33%)	47 (48.45%)	37 (50.00%)	74 (67.89%)
< 60	249 (43.99%)	153 (56.67%)	50 (51.55%)	37 (50.00%)	35 (32.11%)
Unknown	1 (0.18%)	0	0	0	0
Sex					
Male	415 (73.32%)	223 (82.59%)	66 (68.04%)	58 (78.38%)	104 (95.41%)
Female	151 (26.68%)	47 (17.41%)	31 (31.96%)	16 (21.62%)	5 (4.59%)
Smoking					
Yes	423 (74.73%)	222 (82.22%)	NA	59 (79.73%)	108 (99.08%)
No	128 (22.61%)	48 (17.78%)	NA	15 (20.27%)	1 (0.92%)
Unknown	15 (2.65%)	0	NA	0	0
Alcohol					
Yes	371 (65.55%)	239 (88.52%)	NA	NA	58 (53.21%)
No	182 (32.16%)	31 (11.48%)	NA	NA	51 (46.79%)
Unknown	13 (2.3%)	0	NA	NA	0
Tumor site					
Oral cavity	346 (61.13%)	83 (30.74%)	97 (100%)	71 (95.95%)	0
Oropharynx	82 (14.49%)	102 (37.78%)	0	3 (4.05%)	0
Larynx	128 (22.61%)	48 (17.78%)	0	0	109 (100%)
Hypopharynx	10 (1.77%)	33 (12.22%)	0	0	0
Unknown	0	4 (1.48%)	0	0	0
T classification					
T1-T2	218 (38.52%)	115 (42.59%)	NA	30 (40.54%)	NA
T3-T4	344 (60.78%)	155 (57.41%)	NA	44 (59.46%)	NA
Unknown	4 (0.71%)	0	NA	0	NA
N classification					
Negative	295 (52.12%)	94 (34.81%)	NA	42 (56.76%)	NA
Positive	267 (47.17%)	176 (65.19%)	NA	32 (43.24%)	NA
Unknown	4 (0.71%)	0	NA	0	NA
Stage					
I-II	135 (23.85%)	55 (20.37%)	41 (42.27%)	19 (25.68%)	30 (27.52%)
III-IV	417 (73.67%)	215 (79.63%)	56 (57.73%)	55 (74.32%)	79 (72.48%)
Unknown	14 (2.47%)	0	0	0	0
HPV status					
Positive	68 (12.01%)	60 (22.22%)	0	NA	NA
Negative	274 (48.41%)	209 (77.41%)	97 (100%)	NA	NA
Unknown	224 (39.58%)	1 (0.37%)	0	NA	NA
Radiotherapy					
Yes	304 (53.71%)	NA	NA	47 (63.51%)	54 (49.54%)
No	171 (30.21%)	NA	NA	26 (35.14%)	43 (39.45%)
Unknown	91 (16.08%)	NA	NA	1 (1.35%)	12 (11.01%)
Treatment					
Unimodal	188 (33.22%)	78 (28.89%)	43 (44.33%)	25 (33.78%)	43 (39.45%)
Multimodal	278 (49.12%)	189 (70.00%)	53 (54.64%)	48 (64.87%)	54 (49.54%)
Palliative	1 (0.17%)	3 (1.11%)	0	0	0
Unknown	99 (17.49%)	0	1 (1.03%)	1 (1.35%)	12 (11.01%)
CSC gene expression signature					
CSC-HR subgroup	285 (50.35%)	122 (45.19%)	38 (39.18%)	47 (63.51%)	57 (52.29%)
CSC-LR subgroup	281 (49.65%)	148 (54.81%)	59 (60.82%)	27 (36.49%)	52 (47.71%)

^a^*HNSCC* Head and neck squamous cell carcinoma, *TCGA* The Cancer Genome Atlas, *FHCRC* Fred Hutchinson Cancer Research Center, *MDACC* MD Anderson Cancer Center, *CSC* Cancer stem cell, *CSC-HR* CSC gene expression-associated high-risk, *CSC-LR* CSC gene- expression associated low-risk, *NA* Not available

The sensitivity and specificity of the CSC gene expression signatures were identified in each cohort using ROC curves. The AUCs were 0.608, 0.572, and 0.541 for the 1-year, 3-year, and 5-year OS, respectively, in the Leipzig cohort (95% CI, 0.523–0.692, 0.489–0.655, and 0.413–0.668, respectively; Fig. 2b). The AUCs for the 5-year OS were 0.671 and 0.876 in the FHCRC and MDACC cohorts (95% CI, 0.574–0.767 and 0.790–0.960, respectively; Fig. 2d and f), indicating good discriminatory ability in the MDACC cohort [31]. The AUCs for 1-year, 3-year, and 5-year OS were 0.692, 0.628, and 0.538 in the Greece cohort (95% CI, 0.581–0.803, 0.518–0.737, and 0.385–0.691, respectively; Fig. 2h). The AUC for the 5-year OS was 0.582 for all patients in the five cohorts (95% CI, 0.531–0.632; Fig. S3b). These results support the prognostic value of the CSC gene expression signature in the analyzed cohorts.

### The CSC gene expression signature as an independent prognostic factor of non-oropharyngeal HNSCC

To assess the independent prognostic factors of HPV (–) HNSCC patients, we decided to select non-oropharyngeal HNSCC patients in the five independent HNSCC cohorts. The HPV status was missing in many patients, thus we hypothesized that analysis of non-oropharyngeal HNSCC might help find prognostic factors of HPV (-) HNSCC patients. The Greece cohort did not report OS but RFS, so non-oropharyngeal HNSCC patients were selected from other four cohorts (*n* = 816). Cox proportional hazards models using CSC gene expression signatures, patient demographics, alcohol history, smoking history, and clinical staging of non-oropharyngeal HNSCC patient. Upon analysis, the CSC gene expression signature (CSC-HR *vs.* CSC-LR) and regional lymph node metastasis (N + *vs.* N-) were independent prognostic factors of OS in non-oropharyngeal HNSCC patients (*p* = 0.0140 and 0.0292, respectively; Table S2).

### Association of the CSC gene expression signature with HPV status of HNSCC

**Fig. 3:**
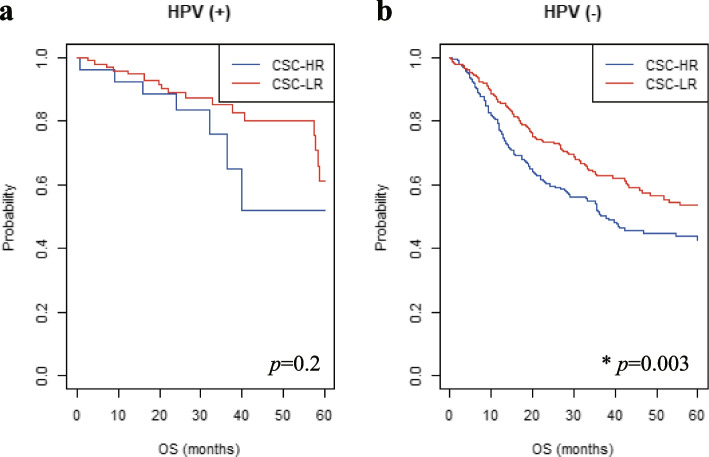
Association of CSC gene expression signature with HPV status in the three independent HNSCC cohorts (TCGA, Leipzig and FHCRC). **a**-**b** The 5-year OS rates for the CSC-HR and CSC-LR subgroups in patients with HPV ( +) and HPV (–) HNSCC were depicted using Kaplan–Meier plots (*n* = 128 and 578; *p* = 0.2 and 0.003, respectively). *p < 0.05

We thought that if the additional survival analysis was performed individually according to HPV status, it might be helpful to find appropriate indications to investigate the CSC gene expression signatures to that can predict patient prognosis in HNSCC. Thus, we analyzed the prognosis of the CSC-HR and CSC-LR subgroups in patients with HPV ( +) and HPV (–) HNSCC from the three HNSCC cohorts (TCGA, Leipzig and FHCRC) that include information about the HPV status (Fig. 3). There were no significant differences in the 5-year OS rates between the CSC-HR and CSC-LR subgroups in patients with HPV ( +) HNSCC (*n* = 128 and *p* = 0.2; Fig. 3a). However, the CSC-HR subgroup showed significantly lower 5-year OS rates than the CSC-LR subgroup, among patients with HPV (–) HNSCC (*n* = 578 and *p* = 0.003; Fig. 3b).

### Association of the CSC gene expression signature with the results of radiotherapy (RT)

**Fig. 4:**
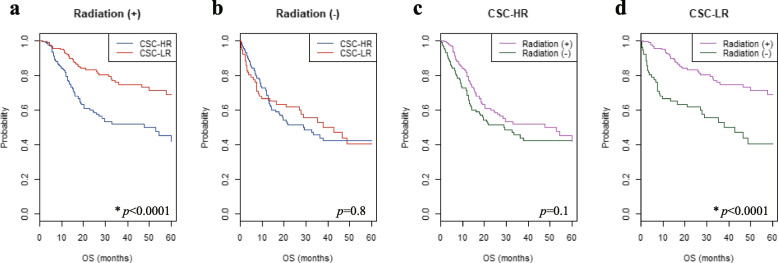
Association of CSC gene expression signature with radiotherapy in the two independent HNSCC cohorts (TCGA and MDACC). **a**-**b** The 5-year OS rates for the CSC-HR and CSC-LR subgroups in patients with HNSCC who did and did not receive radiotherapy (*n* = 348 and 196, respectively) and (**c**, **d**) those for patients with HNSCC who did and did not receive radiotherapy in the CSC-HR (*n* = 293) and CSC-LR (*n* = 251) subgroups were depicted using Kaplan–Meier plots. Patients in the CSC-LR subgroup benefited significantly from radiotherapy (*p* < 0.0001 and < 0.0001, respectively). *p < 0.05

The expression of CSC markers is correlated with poor prognosis after RT in HNSCC [32, 33]. However, the clinical correlation between RT and genes encoding CSC markers has not yet been clearly studied. Thus, we analyzed the prognosis of the CSC-HR and CSC-LR subgroups in the two HNSCC cohorts (TCGA and MDACC) that include information on whether RT has been received or not. The CSC-HR subgroup showed significantly lower 5-year OS rates than the CSC-LR subgroup, among patients with HNSCC who received RT (*p* < 0.0001; Fig. 4a). However, there were no significant differences in the 5-year OS rates between the two subgroups of patients with HNSCC who did not receive RT (Fig. 4b). Similarly, there were no significant differences in 5-year OS rates between patients who received RT and those who did not, in the CSC-HR subgroup (*p* = 0.1; Fig. 4c). However, the prognosis was better in patients who received RT than in those who did not, in the CSC-LR subgroup (*p* < 0.0001; Fig. 4d). To determine any correlation between CSC gene expression signatures and RT in HNSCC, we performed an interaction test for OS. The results revealed a significant correlation between the CSC gene expression signature and RT (*p* < 0.0001).

### Pathway analysis

A total of 8 significant Kyoto Encyclopedia of Genes and Genomes pathways were identified using DAVID (Table S3). Several of these pathways appeared to be related to the cancer or HNSCC pathways, including focal adhesion (*p* = 1.0E-5), small-cell lung cancer (*p* = 3.1E-4), ECM–receptor interaction (*p* = 4.6E-3), proteoglycans in cancer (*p* = 7.2E-3), and PI3K-Akt signaling pathway (*p* = 1.0E-2). Pathways associated with endothelial-mesenchymal transition signaling were also identified, such as regulation of the actin cytoskeleton (*p* = 8.5E-3) and leukocyte transendothelial migration (*p* = 9.9E-3).

## Discussion

In this study, we developed and validated CSC gene expression signatures in five independent HNSCC cohorts. We observed that patients in the CSC-HR subgroup had worse prognosis than those in the CSC-LR subgroup, in each cohort. Similar results were observed in the two subgroups of patients with HPV (–) HNSCC. Furthermore, the CSC gene expression signature could accurately predict the outcomes of patients receiving RT. Thus, the CSC gene expression signature could identify patients with HNSCC who do not respond to RT and require intensified or personalized treatment.

Cancer cells within individual tumor masses often represent distinct phenotypic states that differ in their functional attributes [7]. Within this tumor heterogeneity, CSCs are essential for tumor initiation, maintenance, recurrence, and metastasis. To date, the identification of CSCs has mainly been based on CSC surface markers. However, the genes encoding these CSC biomarkers that could predict the prognosis of patients with HNSCC have not been clearly studied. Thus, we focused on the association between CSC biomarker genes and prognosis of patients with HNSCC.

We believed that it would be difficult to predict the prognosis of HNSCC by considering all CSC biomarker genes, since each CSC biomarker gene had different effects on the prognosis, and the proportion of the expression of each CSC biomarker gene is heterogeneous depending on each patient. Thus, we decided to select CSC biomarker genes that satisfied the following criteria: (a) whose corresponding biomarker expression showed clinical significance in more than two studies in the last 10 years and (b) whose high expression of each gene was significantly associated with prognosis in patients with HNSCC. On the above basis, we selected four CSC biomarker genes, *CD44*, *MET*, *ALDH1A1*, and *BMI1*. We then comprehensively analyzed five independent public cohorts while considering gene signatures associated with these CSC biomarker genes.

CD44 is a transmembrane glycoprotein that is the major receptor for hyaluronan [9]. CD44 is a commonly used CSC marker and is associated with prognosis in various human tumors, including HNSCC [34]. High CD44 expression is associated with poor survival in HNSCC [22]. CD44 is also highly expressed in proliferating cells obtained from N + HNSCC metastasis, thereby highlighting its possible role in tumor progression [23]. In addition, CD44 is a biological factor that is significantly correlated with response to RT, in patients with early stage laryngeal cancer [32].

The expression of c-MET (a mesenchymal-to-epithelial transition factor) was found to be a CSC marker that is positively correlated with the expression of CD44 in HNSCC clinical databases [35]. Lim et al. found that activation of the c-MET pathway is critical for the proliferation and maintenance of CSC traits in HNSCC [36]. c-MET knockdown significantly decreased the expression of CD44-positive cells [36]. c-MET is expressed in the majority of locally advanced HNSCC, and high expression of c-MET predicts a worse prognosis [24]. High MET expression has also been found to be associated with poor loco-regional tumor control and increased metastasis after post-operative chemoradiotherapy in patients with HPV (–) HNSCC [5].

Aldehyde dehydrogenase 1 (ALDH1) and B-lymphoma moloney murine leukemia virus insertion region-1 (BMI-1) are two of the most studied CSC markers in HNSCC [37]. ALDH1 is an important stem cell marker in both normal and cancer cells [38]. ALDH1 regulates cellular functions by detoxifying various aldehydes and retinoid signaling. ALDH1 appears to have protective properties against HNSCC [22]. In another study, the positive expression of ALDH1 showed significant correlation with lymph node metastasis and poor prognosis [25]. The positivity of ALDH1 was also found to be correlated with the number of cells undergoing epithelial-mesenchymal transition and metastasis in early stage oral squamous cell carcinoma (OSCC) [27]. However, the association between ALDH1 expression and prognosis is contradictory.

BMI-1 is important for the self-renewal ability of stem cells and is related to epithelial-mesenchymal transition [39]. Rao et al. found a significant positive correlation between ALDH1 and BMI-1 expression in OSCC tissue samples, although the underlying pathways have not yet been elucidated [25]. High expression of BMI-1 was associated with poor prognosis in advanced-stage HNSCC treated with primary chemoradiotherapy [22]. BMI1 is also upregulated after irradiation in OSCC, and is associated with poor prognosis [28]. Based on these results, the CSC biomarker genes selected in this study may play a significant role in the prognosis of HNSCC.

There are genes, other than CSC genes, whose expression is associated with the diagnosis and prognosis of HNSCC. Lohavanichbutr et al. identified and validated a 13-gene expression signature that was strongly predictive of survival in HPV (–) OSCC patients [15]. They first identified 131 genes by comparing the differential gene expression between OSCC and normal control groups [40]. Thirteen of these genes were then further screened using the L1-penalized Cox proportional hazard regression method. Three genes, *LAMC2, SERPINE1, KLF7*, were found to overlap between the 13 gene expression signatures identified in the study by Lohavanichbutr et al. and the 81 CSC gene expression signatures identified in our analysis. *LAMC2*, *SERPINE1*, *KLF7* play a role in cell proliferation, migration, and adhesion. High expression of these genes is associated with poor prognosis in HNSCC [41–43]. Hypoxia- and ferroptosis-related gene signatures predicting the prognosis of patients with OSCC have also been identified and validated [44, 45]. In this study, we developed and validated signatures associated with CSC biomarker genes, the expression of which was correlated with the prognosis of patients with HNSCC.

Patients with HPV (–) HNSCC have a worse prognosis in terms of OS and RFS rates than those with HPV ( +) HNSCC [46]. However, each patient with HPV (–) HNSCC has a different prognosis owing to various risk factors. We confirmed that the CSC gene expression signature was an independent prognostic factor of non-oropharyngeal HNSCC. Since many non-oropharyngeal HNSCC patients did not include HPV status, we indirectly analyzed the role of CSC gene expression signature in HPV (–) HNSCC using information about non-oropharyngeal HNSCC patients. In addition, the CSC-HR subgroup showed a significantly worse prognosis than the CSC-LR subgroup, among patients with HPV (–) HNSCC. Next, we investigated whether the CSC gene expression signatures influence the prognosis of patients with HPV ( +) HNSCC. In these patients, the 5-year OS rates tended to be lower in the CSC-HR subgroup than in the CSC-LR subgroup; however, the differences were not significant. This may be due to the relatively small size of the HPV ( +) HNSCC cohort (*n* = 128). There is a need for further studies in larger HPV ( +) HNSCC cohorts, to confirm the association between CSC gene expression signatures and prognosis of patients with HPV ( +) HNSCC.

CSCs can regulate their proliferative and self-renewal capacity, and are thus, involved in metastasis, cancer development, and resistance to RT [47]. However, the association between various CSC biomarker genes and the response to RT in HNSCC has not been studied. Only the mRNA expression of CD44 has been shown to be a significant predictor of local recurrence after RT in early stage laryngeal cancer [32]. Thus, we hypothesized that the overexpression of a specific mRNA of CSC biomarker genes in HNSCC might be correlated with response to RT. However, each patient heterogeneously expresses various CSC biomarker genes, and thus, might respond heterogeneously to RT. Our results showed that compared to the CSC-HR subgroup, the CSC-LR subgroup benefited significantly from RT. These results indicated that the CSC gene expression signature might help to program a RT schedule, if further research is conducted on the response to various doses of irradiation in CSC-HR and CSC-LR HNSCC cell lines.

A limitation of our study is that we analyzed CSC gene expression signatures using five different public HNSCC cohorts. Thus, there was a difference in the essential information that was available for each cohort. In particular, the HPV status was missing in about 40% in TCGA cohort and all patients in MDACC and Greece cohorts. Thus, it was not possible to accurately evaluate the effect of the CSC gene expression signature in prognosis of HNSCC patients with HPV (–) status. Instead, we hypothesized that analysis of non-oropharyngeal HNSCC regardless of the HPV status might help find independent prognostic factors of HPV (–) HNSCC patients. In addition, detailed treatment modality methods or doses, such as post-operative RT, concurrent chemoradiotherapy, and induction chemoradiotherapy with surgery, were not included in each cohort. To compensate for the missing information, we conducted an additional analysis on the CSC gene expression signature and found that the CSC gene expression signature was associated with the prognosis of patients with HPV (–) HNSCC and the response to RT in HNSCC. Finally, the mRNA expression of selected CSC biomarker genes showed very low values for AUC as well as sensitivity and specificity that were below the thresholds required for decision-making in clinical settings (AUCs were less than 0.6 for *CD44*, *MET*, *ALDH1A1*, and *BMI1*). A possible reason for the same seems to be that the prognosis of HNSCC is not entirely changed by the mRNA expression of only a single gene, because the cancer is caused by the accumulation of multiple mutations in various pathways. However, these four genes have shown clinically significant association with the expression of corresponding CSC biomarker proteins in HNSCC over the past 10 years [5, 8, 22–28]. Thus, we analyzed and confirmed the actual association between mRNA expression of these genes and prognosis in TCGA HNSCC cohort, by referring to these ROC curves.

To the best of our knowledge, this is the first study to assess the prognosis of patients with HNSCC using various CSC biomarker genes. Each CSC biomarker gene influences the prognosis of patients with HNSCC, but the proportions of these genes are highly heterogeneous in each patient. Thus, we first clarified that the gene expression signatures of the four reference CSC biomarker genes, *CD44*, *MET*, *ALDH1A1*, and *BMI1*, were significantly related to the prognosis of patients with HNSCC. In addition, the Cox proportional hazards model showed that the CSC gene expression signature was an independent prognostic factor that influenced the OS of non-oropharyngeal HNSCC patients.

## Conclusions

We developed CSC gene expression signatures that could predict the prognosis of patients with HNSCC, especially in case with HPV (–) status. CSC gene expression signatures was an independent prognostic factor of non-oropharyngeal HNSCC which mostly indicates HPV (–) status. In addition, there was a significant correlation between the CSC gene expression signature and the response to RT in HNSCC. Therefore, our data provide evidence that CSC gene expression signatures may help in the design of personalized treatments for patients with HPV (–) HNSCC who were classified in more detail.

## Supplementary Information

**Additional file 1: Supplementary Figure 1.** The prognosis according to the mRNA expression of each CSC gene in TCGA cohort. Cut-off values for continuous mRNA expression values were selected while referring to the ROC curve analysis. Kaplan–Meier plots showing the 5-year OS or RFS rates between the two subgroups based on the cut-off values of each CSC gene in TCGA cohort were depicted. Log-rank test was used to compare the prognosis of the two subgroups for each gene, and the plots with the lower p-value between plots depicting the 5-year OS and RFS for each gene were then selected. (a-d) *CD44*, *MET*, *ALDH1A1*, and *BMI1* showed significant differences in the OS or RFS rates between the two subgroups classified according to the mRNA expression in TCGA cohort (*p*=0.0069, 0.0051, 0.028, and 0.021, respectively). (e-g) There were no significant differences in the OS or RFS rates between the two subgroups classified according to the mRNA expression of *PROM1*, *SOX2*, and *POU5F1* in TCGA cohort (*p*=0.11, 0.097, and 0.12, respectively). *p<0.05

**Additional file 2: Supplementary Figure 2.** Construction of the prediction model. (a) Venn diagram showing CSC gene expression signatures correlated with the four CSC genes – *CD44*, *MET*, *ALDH1A1*, and *BMI1*. (b) Schematic overview of the strategy used for constructing the prediction models and evaluating the predicted outcomes based on the CSC gene expression signatures.

**Additional file 3: Supplementary Figure 3.** Kaplan–Meier and ROC analyses for OS of all patients in the five independent cohorts. All patients were classified into CSC-HR and CSC-LR subgroups using the 81 CSC gene expression signatures. (a) Kaplan–Meier plots showing significant difference in the OS rates between the two groups (*p*<0.0001). (b) ROC curves showing the sensitivity and specificity of the CSC gene expression signatures in predicting 1-year, 3-year, and 5-year patient OS in the five independent cohorts (AUC=0.582 for the 5-year OS). *p<0.05

**Additional file 4: Supplementary Table 1.** Eighty-one CSC gene expression signatures in TCGA HNSCC cohort. **Supplementary Table 2.** Univariate and multivariate analyses of the characteristics associated with overall survival in patients with non-oropharyngeal cases in the four independent HNSCC cohorts (*n*=816). **Supplementary Table 3.** A total of 8 significant KEGG pathways associated with CSC gene expression signatures.

## Data Availability

The datasets supporting the conclusions of this study are available from the UCSC Cancer Genomics Browser (https://xena.ucsc.edu/public) (TCGA cohort, *n* = 566) and the National Center for Biotechnology Information Gene Expression Omnibus database (http://www.ncbi.nlm.nih.gov/geo) (Leipzig cohort, GSE65858, *n* = 270; FHCRC cohort, GSE41613, *n* = 97; MDACC cohort, GSE42743, *n* = 74; Greece cohort, GSE27020, *n* = 109).
