# Supplementary figures and images for: Association between cancer stem cell gene expression signatures and prognosis in head and neck squamous cell carcinoma
Source: BMC Cancer. 2022 Oct 19;22:1077. doi: 10.1186/s12885-022-10184-4 (PMC9583594; doi:10.1186/s12885-022-10184-4)

Supplementary Fig. 1

a

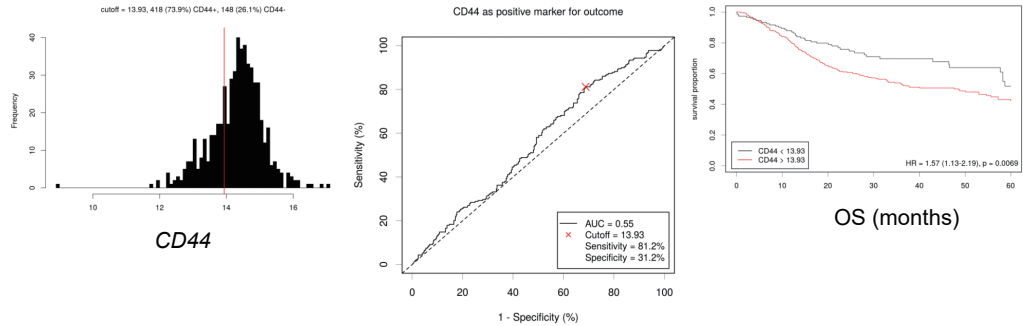

b

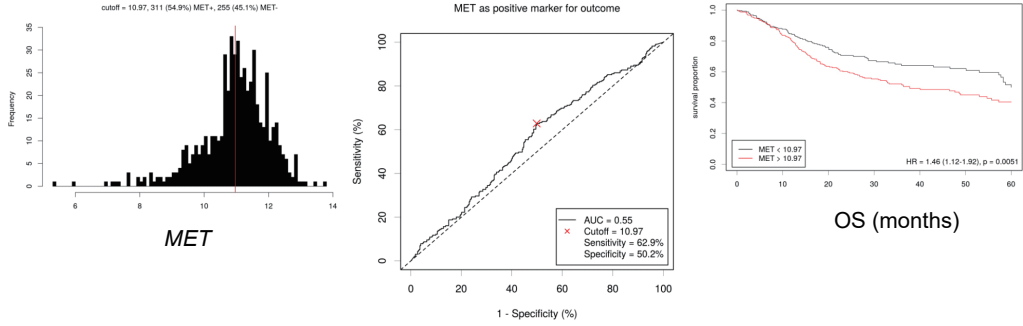

c

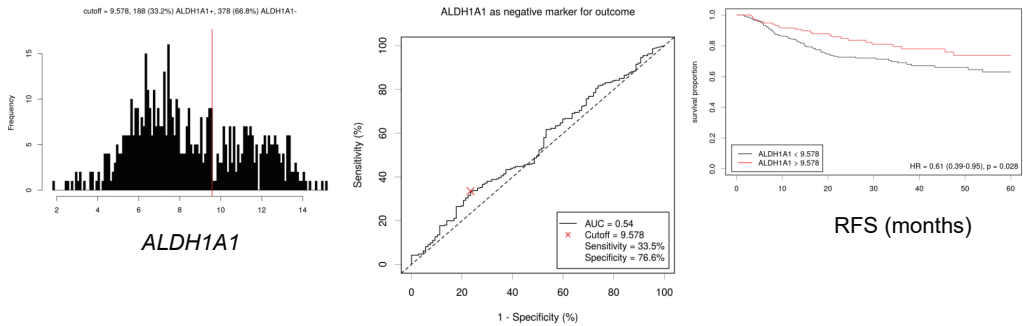

d

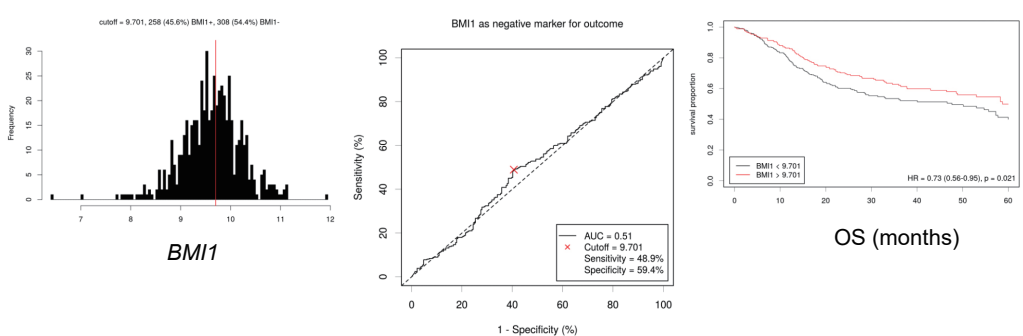

e

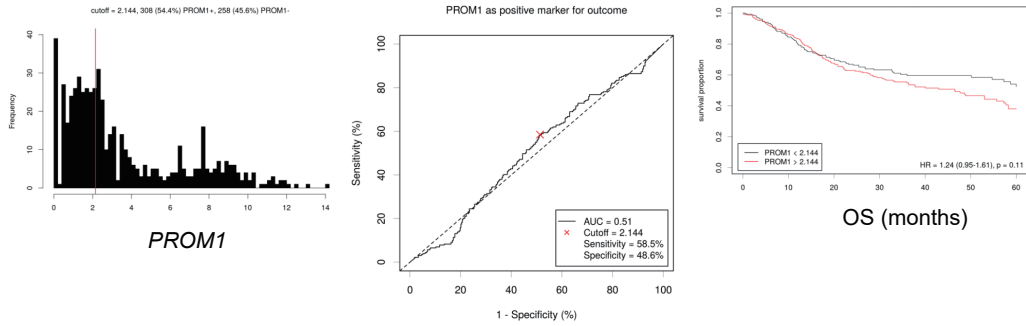

f

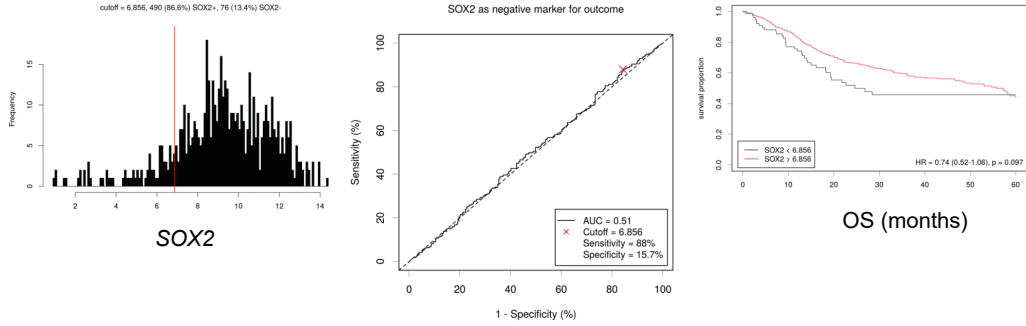

g

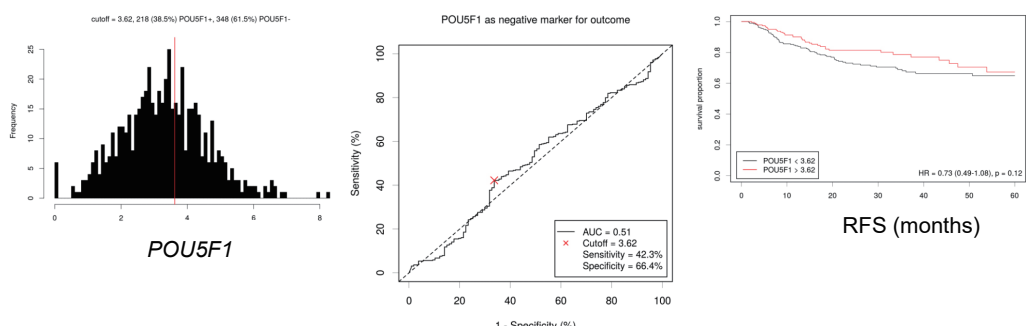

Supplement: Supplementary file 1 — Additional file 1: Supplementary Figure 1. The prognosis according to the mRNA expression of each CSC gene in TCGA cohort. Cut-off values for continuous mRNA expression values were selected while referring to the ROC curve analysis. Kaplan–Meier plots showing the 5-year OS or RFS rates between the two subgroups based on the cut-off values of each CSC gene in TCGA cohort were depicted. Log-rank test was used to compare the prognosis of the two subgroups for each gene, and the plots with the lower p-value between plots depicting the 5-year OS and RFS for each gene were then selected. (a-d) CD44, MET, ALDH1A1, and BMI1 showed significant differences in the OS or RFS rates between the two subgroups classified according to the mRNA expression in TCGA cohort (p=0.0069, 0.0051, 0.028, and 0.021, respectively). (e-g) There were no significant differences in the OS or RFS rates between the two subgroups classified according to the mRNA expression of PROM1, SOX2, and POU5F1 in TCGA cohort (p=0.11, 0.097, and 0.12, respectively). *p<0.05 [file 12885_2022_10184_MOESM1_ESM.pdf]

# Supplementary Fig. 2

a

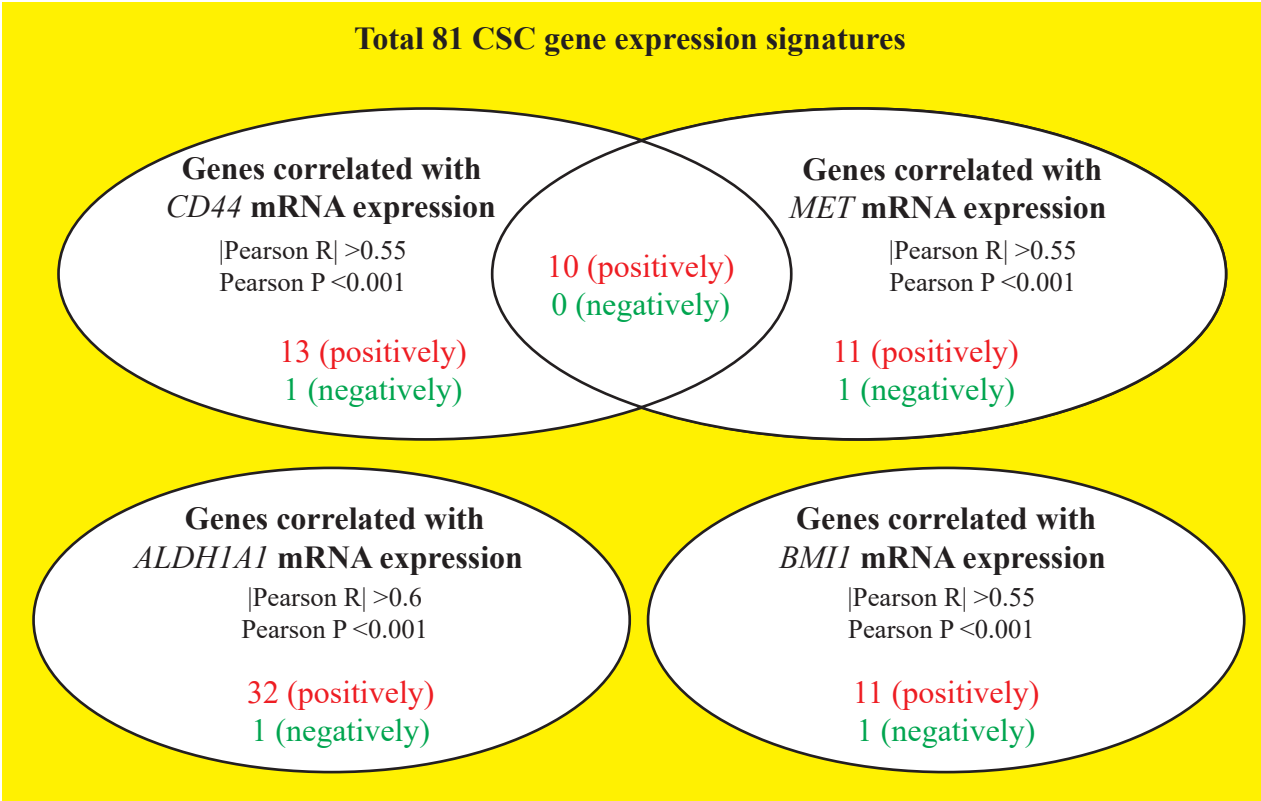

b

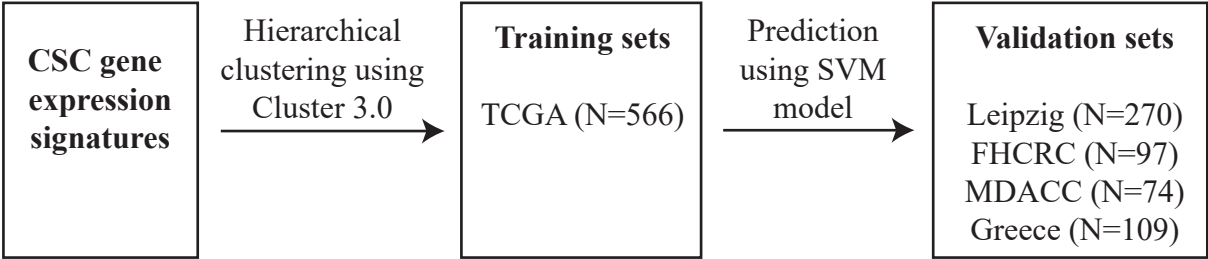

Supplement: Supplementary file 2 — Additional file 2: Supplementary Figure 2. Construction of the prediction model. (a) Venn diagram showing CSC gene expression signatures correlated with the four CSC genes – CD44, MET, ALDH1A1, and BMI1. (b) Schematic overview of the strategy used for constructing the prediction models and evaluating the predicted outcomes based on the CSC gene expression signatures. [file 12885_2022_10184_MOESM2_ESM.pdf]

# Supplementary Fig. 3

a

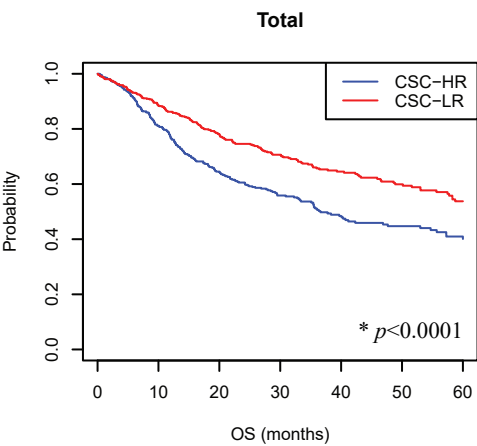

b

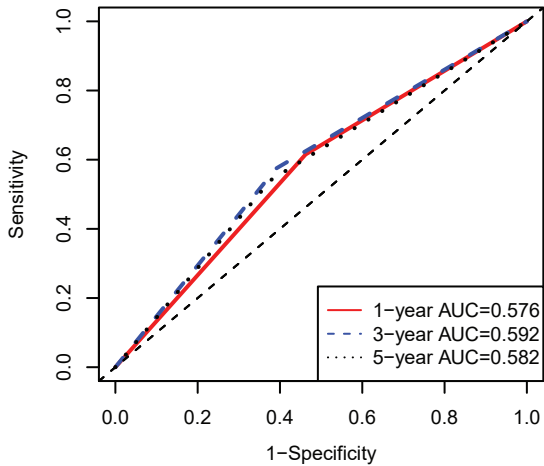

Supplement: Supplementary file 3 — Additional file 3: Supplementary Figure 3. Kaplan–Meier and ROC analyses for OS of all patients in the five independent cohorts. All patients were classified into CSC-HR and CSC-LR subgroups using the 81 CSC gene expression signatures. (a) Kaplan–Meier plots showing significant difference in the OS rates between the two groups (p<0.0001). (b) ROC curves showing the sensitivity and specificity of the CSC gene expression signatures in predicting 1-year, 3-year, and 5-year patient OS in the five independent cohorts (AUC=0.582 for the 5-year OS). *p<0.05 [file 12885_2022_10184_MOESM3_ESM.pdf]
