# Supplementary material for: Association between cancer stem cell gene expression signatures and prognosis in head and neck squamous cell carcinoma
Source: BMC Cancer. 2022 Oct 19;22:1077. doi: 10.1186/s12885-022-10184-4 (PMC9583594; doi:10.1186/s12885-022-10184-4)
Supplement: Supplementary file 4 — Additional file 4: Supplementary Table 1. Eighty-one CSC gene expression signatures in TCGA HNSCC cohort. Supplementary Table 2. Univariate and multivariate analyses of the characteristics associated with overall survival in patients with non-oropharyngeal cases in the four independent HNSCC cohorts (n=816). Supplementary Table 3. A total of 8 significant KEGG pathways associated with CSC gene expression signatures. [file 12885_2022_10184_MOESM4_ESM.docx]

**Supplementary Table 1.** Eighty-one CSC gene expression signatures in TCGA HNSCC cohort.

|  | Pearson’s correlation with gene expression of CD44 | | Pearson’s correlation with gene expression of MET | | Pearson’s correlation with gene expression of ALDH1A1 | | Pearson’s correlation with gene expression of BMI1 | |
| --- | --- | --- | --- | --- | --- | --- | --- | --- |
| Gene symbol | Correlation coefficient | p-value | Correlation coefficient | p-value | Correlation coefficient | p-value | Correlation coefficient | p-value |
| ACTN1  ODZ2  MYO5A  ITGA6  AHNAK2  CDK6  PRNP  F2RL1  PCDHGC5  LIMA1  YPEL1  MYO1B  CAV1  PANX1  MYOF  MSN  CAV2  SNAI2  EXT2  PTHLH  TTPAL  INHBA  BNC1  KLF7  PHLDB2  COL4A6  LAMC2  PXN  SRPK2  ITGB1  SERPINE1  RMND5B  MYO10  XPR1  KIRREL  IRS1  GCNT2  GSTA1  ZDHHC2  NTS  CTTNBP2  SGEF  OXGR1  KIAA1324  TRIM2  PAK7  FAM3B  SLC9A4  LOC730101  MYO5C  ACADSB  SBK1  PLEK2  CHST9  MRAP2  NTRK2  MANSC1  ZBTB7C  SOX2OT  NRXN3  SNX31  SAMD12  ICK  EPHA7  EPS8  SLC16A14  CYP26A1  WNK2  FOXA1  ZMYND11  ZNF124  MAP4K3  ANKRD26  MLLT10  ARHGAP12  TRUB1  CASC4  EPN1  EPC1  SP4  KLHL23 | 0.6328  0.6182  0.6050  0.5994  0.5924  0.5917  0.5908  0.5871  0.5868  0.5805  -0.5801  0.5763  0.5757  0.5755  0.5657  0.5612  0.5605  0.5584  0.5578  0.5555  0.5554  0.5545  0.5537  0.5534 | 1.03E-64  5.26E-61  7.57E-58  1.56E-56  6.06E-55  8.82E-55  1.36E-54  9.22E-54  1.09E-53  2.53E-52  3.00E-52  1.98E-51  2.70E-51  2.89E-51  3.25E-49  2.67E-48  3.65E-48  9.78E-48  1.25E-47  3.69E-47  3.75E-47  5.84E-47  8.17E-47  9.28E-47 | 0.5922  0.5595  0.6303  0.6225  0.5651  0.6012  0.6435  0.6907  0.5758  0.5791  0.6193  0.6079  0.6043  0.5959  0.5899  0.5745  0.5670  -0.5637  0.5578  0.5520  0.5509  0.5505 | 6.59E-55  5.92E-48  4.76E-64  4.35E-62  4.26E-49  6.09E-57  1.59E-67  1.65E-81  2.48E-51  4.92E-52  2.81E-61  1.63E-58  1.16E-57  9.68E-56  2.16E-54  4.78E-51  1.71E-49  8.38E-49  1.24E-47  1.78E-46  2.89E-46  3.53E-46 | 0.6972  0.6958  0.6648  0.6577  0.6525  0.6519  0.6439  0.6427  0.6407  0.6314  0.6277  0.6275  0.6267  0.6234  0.6232  0.6223  -0.6220  0.6217  0.6204  0.6196  0.6175  0.6174  0.6151  0.6114  0.6102  0.6088  0.6077  0.6075  0.6062  0.6060  0.6054  0.6044  0.6027 | 1.20E-83  3.51E-83  1.62E-73  1.84E-71  5.08E-70  7.69E-70  1.23E-67  2.52E-67  8.99E-67  2.45E-64  2.12E-63  2.40E-63  3.80E-63  2.67E-62  3.02E-62  5.02E-62  5.78E-62  6.98E-62  1.48E-61  2.32E-61  7.59E-61  8.31E-61  3.05E-60  2.37E-59  4.58E-59  9.62E-59  1.74E-58  1.95E-58  4.06E-58  4.59E-58  6.32E-58  1.04E-57  2.71E-57 | 0.6431  0.5930  0.5841  0.5805  0.5679  0.5667  0.5608  0.5545  -0.5539  0.5529  0.5520  0.5507 | 2.01E-67  4.48E-55  4.06E-53  2.49E-52  1.15E-49  1.96E-49  3.24E-48  5.82E-47  7.53E-47  1.18E-46  1.74E-46  3.17E-46 |

TCGA, The Cancer Genome Atlas; HNSCC, head and neck squamous cell carcinoma; FC, fold change

**Supplementary Table 2.** Univariate and multivariate analyses of the characteristics associated with overall survival in patients with non-oropharyngeal cases in the four independent HNSCC cohorts (n=816).

| Characteristics | Univariate | | Multivariate (n=707) | |
| --- | --- | --- | --- | --- |
|  | HR (95% CI) | p-value | HR (95% CI) | p-value |
| CSC gene-expression signature  (CSC-HR subgroup) | 1.4844 (1.198-1.84) | 0.0003 * | 1.3367 (1.0605-1.685) | 0.0140 * |
| Age (>60 years) | 1.0868 (0.879-1.344) | 0.442 |  |  |
| Gender (male) | 0.7975 (0.6339-1.004) | 0.0536 |  |  |
| Alcohol (yes) | 0.8575 (0.6596-1.115) | 0.251 |  |  |
| Smoking (yes) | 0.8278 (0.6318-1.085) | 0.171 |  |  |
| Primary tumor (T3 & T4) | 1.3675 (1.071-1.747) | 0.0122 * | 1.4565 (0.9582-2.214) | 0.0784 |
| Regional lymph node (N+) | 1.3532 (1.079-1.697) | 0.0088 * | 1.3587 (1.0315-1.790) | 0.0292 * |
| Stage (stage III & IV) | 1.5853 (1.229-2.046) | 0.0004 * | 0.7789 (0.4615-1.314) | 0.3492 |

HNSCC, head and neck squamous cell carcinoma; HR, hazard ratio; CI, confidence interval; CSC, cancer stem cell; CSC-HR, CSC gene expression-associated high-risk. *p<0.05

**Supplementary Table 3.** A total of 8 significant KEGG pathways associated with CSC gene expression signature.

| KEGG pathway | Gene counts | p-value | Genes |
| --- | --- | --- | --- |
| Focal adhesion | 8 | 1.0E-5 * | ACTN1, CAV1, CAV2, COL4A6, ITGA6, ITGB1, LAMC2, PXN |
| Small cell lung cancer | 5 | 3.1E-4 * | COL4A6, CDK6, ITGA6, ITGB1, LAMC2 |
| Bacterial invasion of epithelial cells | 4 | 3.4E-3 * | CAV1, CAV2, ITGB1, PXN |
| ECM-receptor interaction | 4 | 4.6E-3 * | COL4A6, ITGA6, ITGB1, LAMC2 |
| Proteoglycans in cancer | 5 | 7.2E-3 * | CAV1, CAV2, ITGB1, MSN, PXN |
| Regulation of actin cytoskeleton | 5 | 8.5E-3 * | ACTN1, ITGA6, ITGB1, MSN, PXN |
| Leukocyte transendothelial migration | 4 | 9.9E-3 * | ACTN1, ITGB1, MSN, PXN |
| PI3K-Akt signaling pathway | 6 | 1.0E-2 * | COL4A6, CDK6, IRS1, ITGA6, ITGB1, LAMC2 |

CSC, cancer stem cell. *p<0.05
